# Antibiotic Prescriptions in Critically Ill Patients with Bloodstream Infection Due to ESBL-Producing Enterobacteriaceae: Compliance with the French Guidelines for the Treatment of Infections with Third-Generation Cephalosporin-Resistant Enterobacteriaceae—A Multicentric Retrospective Cohort Study

**DOI:** 10.3390/microorganisms11112676

**Published:** 2023-10-31

**Authors:** Camille Le Berre, Marion Houard, Anne Vachée, Hugues Georges, Frederic Wallet, Pierre Patoz, Patrick Herbecq, Saad Nseir, Pierre-Yves Delannoy, Agnès Meybeck

**Affiliations:** 1 Service de Réanimation et Maladies Infectieuses, Centre Hospitalier de Tourcoing, 135 Rue du Président Coty, 59200 Tourcoing, France; camille.leberre95@gmail.com (C.L.B.); hgeorges@ch-tourcoing.fr (H.G.); pydelannoy@ch-tourcoing.fr (P.-Y.D.); 2 Service de Réanimation Médicale, CHRU de Lille, 2 Avenue Oscar Lambret, 59000 Lille, France; marion.houard@chru-lille.fr (M.H.); saad.nseir@chru-lille.fr (S.N.); 3 Laboratoire de Microbiologie, Centre Hospitalier de Roubaix, 11 Boulevard Lacordaire, 59100 Roubaix, France; anne.vachee@ch-roubaix.fr; 4 Laboratoire de Microbiologie, CHRU de Lille, 2 Avenue Oscar Lambret, 59000 Lille, France; frederic.wallet@chru-lille.fr; 5 Laboratoire de Microbiologie, Centre Hospitalier de Tourcoing, 135 Rue du Président Coty, 59200 Tourcoing, France; ppatoz@ch-tourcoing.fr; 6 Service de Réanimation, Centre Hospitalier de Roubaix, 11 Boulevard Lacordaire, 59100 Roubaix, France; patrick.herbecq@ch-roubaix.fr

**Keywords:** extended-spectrum beta-lactamase-producing Enterobacteriaceae, carbapenem sparing, guidelines, antibiotics, critical care, bacteremia

## Abstract

National and international guidelines were recently published regarding the treatment of Enterobacteriaceae resistant to third-generation cephalosporins infections. We aimed to assess the implementation of the French guidelines in critically ill patients suffering from extended-spectrum β-lactamase-producing Enterobacteriaceae bloodstream infection (ESBL-E BSI). We conducted a retrospective observational cohort study in the ICU of three French hospitals. Patients treated between 2018 and 2022 for ESBL-E BSI were included. The primary assessment criterion was the proportion of adequate empirical carbapenem prescriptions, defined as prescriptions consistent with the French guidelines. Among the 185 included patients, 175 received an empirical anti-biotherapy within 24 h of ESBL-E BSI onset, with a carbapenem for 100 of them. The proportion of carbapenem prescriptions consistent with the guidelines was 81%. Inconsistent prescriptions were due to a lack of prescriptions of a carbapenem, while it was recommended in 25% of cases. The only factor independently associated with adequate empirical carbapenem prescription was ESBL-E colonization (OR: 107.921 [9.303–1251.910], *p* = 0.0002). The initial empirical anti-biotherapy was found to be appropriate in 83/98 patients (85%) receiving anti-biotherapy in line with the guidelines and in 56/77 (73%) patients receiving inadequate anti-biotherapy (*p* = 0.06). Our results illustrate the willingness of intensivists to spare carbapenems. Promoting implementation of the guidelines could improve the proportion of initial appropriate anti-biotherapy in critically ill patients with ESBL-E BSI.

## 1. Introduction

Extended-spectrum β-lactamase-producing Enterobacteriaceae bloodstream infection (ESBL-E BSI) can lead to high morbidity and mortality, particularly when initial antibiotic treatment is inappropriate [1,2]. The choice of empirical antimicrobial therapy is crucial, especially in patients with septic shock. The reference treatment for ESBL-E infections is carbapenem. As a result of the widespread use of carbapenems, the rise in carbapenem-resistant Enterobacteriaceae (CRE) has justified carbapenem-sparing strategies [3]. Piperacillin−tazobactam (PIP-TAZ) is the best alternative to carbapenems in the case of ESBL-E infections [4]. In 2018, the results of a multicenter randomized MERINO clinical trial comparing definitive treatment with PIP-TAZ to meropenem in patients with ESBL-E BSI were published. The study failed to prove non-inferiority [5]. In this context, the French National Authority for Health (HAS), the French Infectious Diseases Society (SPILF), and the French Intensive Care Society (SRLF) jointly developed up-to-date guidance on the treatment of suspected and documented infections of Enterobacteriaceae resistant to third-generation cephalosporins (3GCR-E), including ESBL-E [6]. These guidelines aim to improve the use of carbapenems, reducing their consumption to only cases where an empirical or documented prescription is strictly necessary to ensure their effectiveness. The objective of our study was to assess the implementation of these recommendations in critically ill patients suffering from ESBL-E BSI.

## 2. Patients and Methods

### 2.1. Setting and Study Population

We carried out a multicenter retrospective cohort study in three hospitals in northern France. All consecutive patients treated for ESBL-E BSI in the ICUs of Lille, Roubaix, and Tourcoing Hospitals from 1 October 2018 to 1 June 2022 were included. Cases were identified using an analysis of the laboratory database and ICU clinical files. When there were multiple episodes of ESBL-E BSI, we only assessed the first one and considered any subsequent episodes to be recurrences or reinfections. The Vitek 2 system (bioMérieux^®^, Marcy l’Etoile, France) was used for Enterobacteriaceae identification and antibiotic susceptibility testing, following the European Committee on Antimicrobial Susceptibility Testing (EUCAST) guidelines [7].

### 2.2. Data Collection

Upon ICU admission, we recorded the demographic characteristics; reason for admission; comorbidities, especially, immunodeficiency; and severity of illness. McCabe and Jackson criteria were used to classify comorbidities [8]. Immunodeficiencies included neoplasia, neutropenia, any immunosuppressive treatment, and AIDS. Two scores were used to assess the severity of the illness. The Simplified Acute Physiology Score II (SAPS II) was used to estimate the risk of in-hospital death [9]. The Sepsis-Related Organ Failure Assessment (SOFA) score was used to assess organ failure [10]. At the time of BSI onset, we recorded risk factors for ESBL infection, including antibiotics exposure within three months before BSI, nosocomial infection, and prior ESBL-E colonization. Data on duration of hospital and ICU stay, severity of illness, and presence of shock were collected. The usual criteria were used to define shock [11].

Antibiotic prescriptions were recorded. Empirical treatment refers to the use of antibiotics prior to receiving the results from culture and susceptibility testing. It was considered appropriate when the isolated bacteria were susceptible in vitro to at least one of the antibiotics prescribed using the EUCAST breakpoints and when it was started less than 24 h from BSI onset. Definitive treatment refers to the use of antibiotics after receiving the results from the culture and susceptibility testing. De-escalation was defined as switching from combination to monotherapy, or as selecting a definitive beta-lactam with a narrower spectrum and selective pressure [12]. All our patients were followed up until they died or were released from the ICU.

### 2.3. Objectives and Definitions

The main objective of our study was to assess the proportion of adequate empirical antibiotic treatment in ICU patients suffering from ESBL-E BSI, defined as prescriptions consistent with the French guidelines for the treatment of suspected and documented 3GCR-E infections.

The primary judgment criterion was the proportion of empirical carbapenem prescriptions consistent with the French guidelines. An empirical carbapenem prescription was considered adequate in the case of:

Community-acquired pyelonephritis or complicated urinary tract infection (UTI):

- In the absence of septic shock and a history of ESBL-E resistant to PIP-TAZ urinary infection/colonization <3 months;
- In the presence of septic shock and a history of ESBL-E urinary infection/colonization or antibiotic treatment within 3 months.

Hospital-acquired pyelonephritis or complicated UTI:

- A history of ESBL-E urinary infection/colonization or antibiotic treatment within 3 months.

Hospital-acquired intra-abdominal infection:

- Treatment with PIP-TAZ or a cephalosporin active against *P. aeruginosa* within 1 month;
- A history of ESBL-E or PIP-TAZ resistant *P. aeruginosa* infection/colonization within 3 months.

Hospital-acquired pneumonia:

- In the case of ESBL-E colonization:

And severity signs;

Or immunodepression;

Febrile aplasia.

- In the case of septic shock and a history of ESBL-E colonization/infection within 3 months.

As a result of the retrospective nature of our study, a history of travel in endemic areas within 3 months was not considered a risk factor for ESBL-E, as this information was not fully documented in medical records.

The secondary objectives of our study were:

To determine factors associated with an empirical carbapenem prescription consistent with the French guidelines.

To determine the proportion of appropriate initial antibiotic treatments depending on the adequation with the French guidelines for the treatment of 3GC-RE infections. An appropriate anti-biotherapy was defined as the prescription of at least one drug active against ESBL-E isolated in blood cultures. An adequate anti-biotherapy was defined as a choice of antibiotics prescribed in accordance with the recommendations, including the choice of beta-lactam (3CG, PIP-TAZ, carbapenem), and the use of a combination with an aminoglycoside. A prescription of an aminoglycoside was recommended in the case of a severe hospital-acquired infection or in the case of hospital-acquired pneumonia with a history of ESBL-E colonization and an empirical prescription of a non-carbapenem-containing regimen. A severe infection was defined by the presence of septic shock or life-threatening organ dysfunction.

To determine the proportion of adequate definitive antibiotic treatments, especially the proportion of adequate prescriptions of an alternative to carbapenem, defined as prescriptions consistent with the French guidelines. If the evolution was favorable, an adequate definitive prescription of an alternative to carbapenem was made in the case of:

- Acute pyelonephritis or complicated UTI with a susceptible strain in order of preference: trimethoprim–sulfamethoxazole, fluoroquinolone, cefoxitin (in case of *E. coli*), temocillin, amoxicillin–clavulanate, PIP-TAZ, and aminoglycosides;
- Intra-abdominal infection with a controlled source of infection and a strain with PIP-TAZ CMI ≤ 4: PIP-TAZ;
- Pneumonia and a strain with PIP-TAZ CMI ≤ 4: PIP-TAZ; otherwise, if susceptibility to quinolone: a fluoroquinolone. The use of temocillin or trimethoprim–sulfamethoxazole could be proposed.

### 2.4. Statistical Analysis

Quantitative variables are presented in terms of mean and standard deviation, or in terms of median and interquartile interval, based on the normality of their distribution. Student’s test or the Mann–Whitney U test, when appropriate, was used to compare the quantitative variables. Qualitative variables were presented in terms of numbers and percentages. The chi-square test or Fisher’s test, when appropriate, was used to compare the qualitative variables. Differences between groups with a *p*-value of ≤ 0.05 were considered statistically significant. To determine the independent effect of the variables on adequation with the French guidelines for carbapenem prescriptions, we performed a logistic regression analysis, where the multivariate model included all the covariates with *p* < 0.2 in the unadjusted model. We used SAS 9.2 to carry out all the statistical analyses.

## 3. Results

### 3.1. Demographic and Clinical Data

During the study period, 186 patients were diagnosed with ESBL-E BSI. In one case, the medical file was missing. Table 1 summarizes the demographic and clinical characteristics of the 185 patients included in our cohort. The male sex was predominant (68%). The median age was 60.0 years (IQR, 17.8). The type of admission was mainly medical admission (83%). The median SAPS II was 49.0 (IQR, 27.8) at ICU admission. More than one-third of our patients were suffering from diabetes, and 24% were immunocompromised. All our patients except three (98%) had at least one risk factor for ESBL-E infection among antibiotic treatments (83%), ESBL-E colonization within 3 months (79%), or the nosocomial nature of the infection (90%). The most common source of bacteremia was pneumonia in 103 cases (56%), followed by a UTI in 26 cases (14%), a catheter infection in 24 cases (13%), and an abdominal infection in 24 cases (13%).

### 3.2. Microbiological Data

*Klebsiella* sp. (n = 126, 68%), *Escherichia coli* (n = 29, 16%), and *Enterobacter* sp. (n = 22, 12%) were the most frequently involved pathogens (Table 2). The bacteremia was polymicrobial in 15 cases (8%). Regarding antimicrobial susceptibility, all ESBL-E were susceptible to imipenem and meropenem, while only 83% of the strains were susceptible to ertapenem. A total of 54 isolates (29%) were susceptible to PIP-TAZ, with MIC ≤ 4 mg/L in 25 cases (14%). When tested, 95% (114/120) of the isolates were susceptible to ceftazidime–avibactam. Most ESBL-E were resistant to at least a fluoroquinolone (71%) and trimethoprim–sulfamethoxazole (78%). Most strains were susceptible to amikacin (86%). A total of 81 out of the 95 (85%) tested strains were susceptible to colistin, while only 47 out of the 83 tested strains (57%) were susceptible to temocillin.

### 3.3. Empirical Antibiotic Treatment

**Figure 1:**
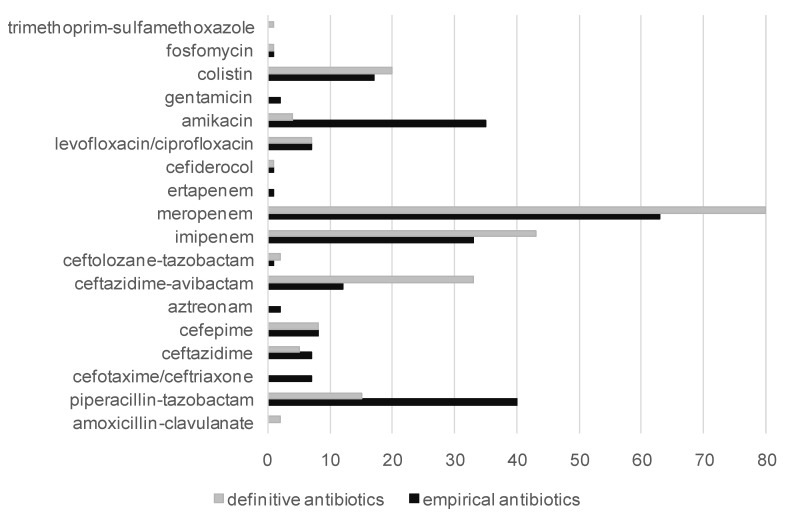
Empirical and definitive antibiotic prescriptions in patients treated for ESBL-E BSI in the ICU.

A total of 175 patients (95%) received empirical antibiotics within 24 h of ESBL-E BSI onset. Empirical treatment contained a carbapenem in 100/175 antibiotic regimen (57%). The other beta-lactams prescribed were mainly PIP-TAZ in 40 patients (23%), ceftazidime–avibactam in 12 patients (7%), cefepime in 8 patients (5%), and ceftazidime in 7 patients (4%). Forty-two patients (24%) were treated with a combination therapy including an aminoglycoside in 36 cases (21%) and a fluoroquinolone in 6 cases (3%). Figure 1 summarizes antibiotics used in the empirical and definitive regimens.

### 3.4. Adequation and Appropriateness of Empirical Antibiotic Treatment

**Figure 2:**
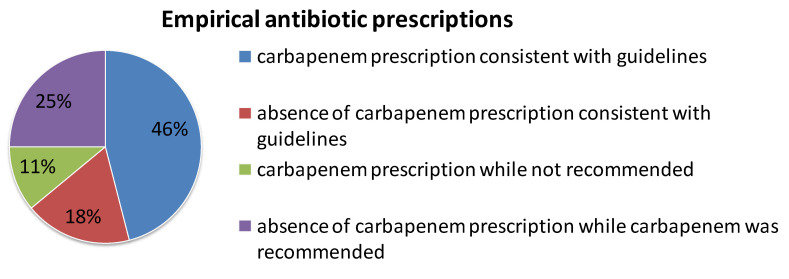
The proportion of empirical carbapenem prescriptions concordant with the French guidelines.

The choice of beta-lactam was in accordance with the guidelines in 111 patients (63%). Regarding the 100 empirical carbapenem prescriptions, 81 (81%) were consistent with the guidelines. Figure 2 shows the proportion of empirical carbapenem and non-carbapenem prescriptions according to their concordance with the French guidelines. In 25% of the cases, an empirical carbapenem was not prescribed even though it was recommended. The choice of a monotherapy or a combination with an aminoglycoside was concordant with the guidelines in 102 patients (58%). Finally, the initial anti-biotherapy was in total adequation with the guidelines in 98 patients (56%).

The initial empirical antibiotic treatment was appropriate in 139 patients (79%): 83 out of 98 (85%) received antibiotic treatment adequate with the guidelines, and 56 out of 77 (73%) received inadequate antibiotic treatment (*p* = 0.06).

### 3.5. Factors Associated with an Empirical Prescription of a Carbapenem in Adequation with the Guidelines

The significant factors associated with an adequate empirical prescription of a carbapenem are detailed in Table 3. In the univariate analysis, chronic respiratory insufficiency (OR: 0.328 [0.109–0.990], *p* = 0.048), ESBL-E colonization (OR: 54.312 [10.192–289.417], *p* <0.0001), SOFA at BSI onset (OR: 54.312 [10.192–289.417], *p* = 0.008), septic shock (OR: 3.885 [1.334–11.313], *p* = 0.013), and respiratory source of infection (OR: 3.152 [1.087–9.134], *p* = 0.035) were the factors associated with an adequate empirical prescription of a carbapenem. In the multivariate analysis, ESBL-E colonization appeared as the only independent factor for the adequation of an empirical carbapenem prescription (OR: 107.921 [9.303–1251.910], *p* = 0.0002).

### 3.6. Definitive Antibiotic Treatment and Adequation with the Guidelines

A total of 182 patients (98%) received a definitive antibiotic treatment, with a carbapenem in 123 cases (68%) (Figure 2). The other beta-lactams prescribed were mainly beta-lactam/beta-lactamase inhibitor combinations (BL/BLI), with ceftazidime–avibactam in 22 patients (12%) and PIP-TAZ in 15 patients (8%). The third-generation cephalosporins used as a definitive antibiotic treatment were cefepime in eight patients (4%) and ceftazidime in five patients (3%). Among our patients, 20 (11%) received colistin, 5 (3%) received an aminoglycoside, and 7 (4%) a fluoroquinolone. However, only one patient received amikacin monotherapy, and two patients received fluoroquinolone monotherapy as a non-carbapenem alternative treatment for UTI. Fluoroquinolone monotherapy was also prescribed in one patient with a catheter-related infection. PIP-TAZ was prescribed in patients with pneumonia (n = 8), a catheter-related infection (n = 4), a UTI (n = 2), and an intra-abdominal infection (n = 1). ESBL-E MIC was ≤4 mg/L in eight cases, 8 mg/L in three cases, and 16 mg/L in three cases. When prescribed, definitive treatment with PIP-TAZ was in accordance with the guidelines in 8 out of 15 patients (53%).

In accordance with French guidelines for the treatment of 3GCR-E infections, an alternative to carbapenem could have been prescribed with clinical improvement in 65 cases (36%), fluoroquinolone in 36 cases (20%), temocillin in 35 cases (19%), PIP-TAZ in 25 cases (14%), trimethoprim–sulfamethoxazole in 25 cases (14%), an aminoglycoside in 20 cases (11%), and cefoxitin in 9 cases (5%). Trimethoprim–sulfamethoxazole and temocillin were the only alternative in 25 cases of pneumonia.

Finally, in our cohort, a non-carbapenem-containing regimen was prescribed as a definitive antibiotic treatment in 54 patients. Its prescription was in accordance with the French guidelines for the treatment of suspected 3GCR-E in 12 out of 54 prescriptions (22%).

## 4. Discussion

In our patients treated for an ESBL-E BSI in the ICU, the prescription of an empirical carbapenem-containing regimen or not was in line with the French guidelines for the treatment of suspected 3GCR-E infections in two-thirds of the cases. An inadequate initial carbapenem prescription was most often related to the lack of a prescription of a carbapenem when it was expected, which occurred in a quarter of the cases. Regarding definitive antibiotic treatment, an alternative to carbapenem was prescribed in almost one-third of the cases, mainly ceftazidime–avibactam or PIP-TAZ. Our results illustrate the willingness of intensivists to spare carbapenems.

All our patients except three had at least one risk factor for ESBL-E infection. The incidence of community-acquired infection with ESBL-E has recently increased [13,14]. Lee CH et al. validated a predictive score for community-onset ESBL-E BSI [15]. In our population of critically ill patients, the majority of cases of ESBL-E BSI were hospital-acquired, with only 10% being community-acquired infections. Other risk factors for ESBL-E were antibiotic exposure or ESBL-E colonization or infection within three months in around 80% of the patients. These factors have been associated with ESBL-E infection occurrence in multiple studies, but none of these risk factors appear to be discriminant in identifying 3GCR-E bacteremia [16]. Taking into account the presence of only one risk factor will lead to a dramatic overuse of carbapenem empirically. Several ESBL-E infection prediction scores have been validated. However, even the most accurate one has a moderate discriminatory ability, leading to an underestimation of the risk of ESBL-E infection [17]. Elligsen M et al. created an algorithm to predict resistance that accounts for previous microbiological results. Its prospective use spared broad-spectrum antibiotic prescriptions with similar proportions of appropriate antimicrobial therapy [18,19].

The choice of empirical treatment cannot be determined only by the presence of risk factors or the calculation of predictive scores. Several national and international guidelines were recently edited regarding the antibiotic treatment of 3GC-RE infections, including ESBL-E infections [6,20,21]. Most of them are limited to the treatment of proven 3GCR-E infections. They detailed the preferred and alternative antibiotic treatments to be used after receiving results from culture and susceptibility testing. The Infectious Diseases Society of America (IDSA) guidelines for the treatment of ESBL-E, CRE, and multi-drug resistant *Pseudomonas aeruginosa* only briefly evoke empirical treatment [20]. The expert panel stated that the choice of empirical treatment should be based on the presumed pathogens, the presence of severity criteria, the origin of the infection, and individualized criteria including antibiotic allergy and renal insufficiency. The cultures and susceptibility testing results in the last 6 months, antibiotic treatments in the last month, and local microbiological epidemiology should also be taken into account. The prevalence of pathogens resistant to all first-line agents is of particular clinical relevance [22]. The selection of an appropriate empirical antibiotic treatment could be improved with the application of emerging concepts relative to predictive microbiology such as escalation antibiogram or regional cumulative antibiogram [23,24]. The French guidelines specifically detail the empirical antibiotic treatment of suspected infections with 3CGR-E depending on the site and the severity of the infection [6]. In our ICU cohort of patients with ESBL-E BSI, the most frequent site of infection was pneumonia, accounting for more than half of the cases. Most of our patients were under mechanical ventilation, and half of them had septic shock at the time of ESBL-E BSI onset. One-quarter of our patients were immunocompromised. Immunosuppression, including HIV infection, has an impact on the incidence, nature, and etiologies of bacteremia [25,26].

Almost all our patients (95%) received empirical antibiotics within 24 h of ESBL-E BSI onset. The Surviving Sepsis Campaign aimed to reduce mortality due to sepsis by improving its early recognition and treatment [27]. Evidence-based guidelines for sepsis management have been developed. The administration of broad-spectrum antibiotics is part of the hour-1 bundle. Models including various clinical data and biochemical and hematologic biomarkers were developed to predict bacteremia [28]. These models could be complementary with traditional C-reactive protein and procalcitonin for guiding antibiotic use [29]. A carbapenem-containing regimen was prescribed in 57% of our patients, while it was recommended, according to the French guidelines, in 71% of the cases. In our cohort, the only factor independently associated with a carbapenem prescription in adequation with the guidelines was a history of ESBL-E colonization. It seemed that the intensivists gave more importance to a prior ESBL-E colonization than to a recent antibiotic exposure when choosing a carbapenem-containing regimen. In a worldwide sample of ICU patients, the prevalence of antibiotic exposure was 71% [30]. Treating empirically all patients with recent antibiotic exposure and suffering from a severe infection in the ICU with a carbapenem will lead to a large amount of prescriptions. French experts selected 29 studies that identified previous antibiotic treatment as a risk factor for ESBL-E infection with an odds ratio from 1.5 to 15.3 [6]. None of these studies included exclusively ICU patients. On the contrary, the impact of ESBL-E colonization on the occurrence of infection with ESBL-E has been specifically assessed in the ICU [31,32]. The rate of ESBL-E ventilator-associated pneumonia was significantly higher in patients colonized with ESBL-E and varied from 22 to 42.5%.

In our cohort, empirical treatment with a BL/BLI combination was prescribed in 30% of the patients as PIP/TAZ (23%) or ceftazidime/avibactam (7%). Ceftazidime/avibactam is a new BL/BLI combination. It has been approved and associated with metronidazole for treating complicated intra-abdominal infections. Its prescription has also been approved in the case of complicated UTI and nosocomial infections, including ventilator-associated pneumonia [33]. Of note, the representation of critically ill patients in the published studies was low [34]. The French guidelines recommend not using it as an empirical treatment in the case of suspected ESBL-E infection. Like other panels, French experts recommended that the new BL/BLI should be reserved for XDR pathogens, such as carbapenem-resistant organisms [6,20,21,35]. PIP/TAZ remains the most extensively studied carbapenem-sparing option. Several retrospective studies and meta-analyses supported its prescription in patients with ESBL-E BSI [36,37]. Recently, the multicenter randomized MERINO clinical trial comparing definitive treatment with PIP/TAZ to meropenem in patients with ESBL-E BSI failed to prove non-inferiority [5]. Its use is now controversial. The French guidelines recommend its empirical prescription in suspected ESBL-E infections mainly in association with amikacin [6].

Apart from the BL/BLI combination, the most-studied combinations for empirical treatment of ESBL-E infections are aminoglycosides in combination with beta-lactams. Expected benefits of these combinations are synergistic bactericidal activity and antimicrobial spectrum broadening, leading to a higher proportion of appropriate initial anti-biotherapy and potential survival improvement, especially in the case of septic shock [38,39]. However, renal toxicity remains an issue. The French guidelines for the treatment of 3GCR-E infections recommend empirical prescription of an aminoglycoside in the case of severe hospital-acquired infection defined by the presence of septic shock, a life-threatening organ dysfunction, or in case of hospital-acquired pneumonia with a history of ESBL-E colonization and empirical prescription of a non-carbapenem regimen [6]. Only one-quarter of our patients treated for an ESBL-E BSI in the ICU received a combination with an aminoglycoside despite septic shock in more than half of them. Isolated strains were susceptible to amikacin in 86% of the cases.

In our cohort, the initial antibiotic treatment was totally consistent with the French guidelines in 56% of the patients, resulting in an appropriate empirical antibiotic treatment of 85%. In the case of inadequate initial anti-biotherapy, the proportion of appropriate therapy was only 73%. Promoting the implementation of the guidelines could improve the proportion of empirically appropriate antibiotic treatments in critically ill patients with ESBL-E BSI.

Regarding definitive antibiotic treatment, the majority of our patients received a carbapenem-containing regimen, amounting to 68% of the cases. The most frequently definitive non-carbapenem treatment was a BL/BLI combination in 20% of our patients, with ceftazidime/avibactam being the most frequently used combination. The French and international guidelines maintain a carbapenem (imipenem or meropenem) as a preferred treatment for patients with ESBL-E BSI or severe infection [6,20,21]. They recommended against the use of ceftazidime–avibactam and ceftolozane–tazobactam as an alternative to carbapenem in order to preserve their effectiveness on carbapenem-resistant bacteria. Once patients are stabilized, the guidelines suggest step-down targeted therapy following carbapenems, using the old BL/BLI, quinolones, trimethoprim–sulfamethoxazole, or other alternatives depending on the susceptibility testing results. PIP-TAZ is recommended for the treatment of UTI by IDSA [20]. Its use is supported by the French guidelines also for non-UTI, if the PIP-TAZ MIC of the isolate is ≤ 4 mg/L and if PIP-TAZ is used as extended or constant infusion [6]. In our cohort, definitive treatment with PIP-TAZ was consistent with the French guidelines in 8 out of 15 cases. In all cases, a continuous infusion was used. Temocillin is another alternative beta-lactam recommended by the French guidelines in the case of UTI and pneumonia. It was not prescribed in our cohort despite being a suggested non-carbapenem regimen according to the guidelines in 19% of our cases. The lack of consistent data about its use in critically ill patients with the exception of pharmacodynamic data justifies the reluctance of physicians to prescribe it in severe ESBL-E infections [40]. Two randomized studies are underway [41,42]. In our cohort, non-beta-lactam alternatives to carbapenems were not used with the exception of one prescription of amikacin and three prescriptions of fluoroquinolone. Data on fluoroquinolone and trimethoprim sulfamethoxazole efficacy for the treatment of ESBL-E BSI are lacking [43,44].

Our study has several limitations. We conducted a multicentric retrospective observational study. All participating centers were located in northern France, which prevents the extension of our results to another region of the world with different ecologies and antibiotic strategies. However, in our patients suffering from ESBL-E BSI, the most frequently isolated ESBL-E microorganisms were *Klebsiella* sp. followed by *Enterobacter* sp. and *Escherichia coli*. These results are in accordance with epidemiological observations in France and abroad [45,46]. Our study was partly conducted during the COVID-19 pandemic, which may have influenced the results. The pandemic has impacted the pattern of BSI, especially in the ICU [47]. We did not assess the impact of new tools, such as syndromic multiplex PCR, which was implemented during the pandemic [48]. The retrospective nature of this study prevented us from taking into account other factors that impact empirical antibiotic prescription in the ICU such as coincident outbreaks with carbapenem-resistant microorganisms [49]. Due to the observational approach, factors related to the physicians, patients, and type of infection were not controlled. Finally, our sample was relatively small, which did not allow for the assessment of the prognostic impact of empirical treatment adequation with the guidelines.

## 5. Conclusions

In our cohort of critically ill patients, empirical carbapenem prescriptions were fewer than expected following the French guidelines for the treatment of 3GCR-E, reflecting the efforts of intensivists to promote carbapenem-sparing regimens. Application of the guidelines tended to increase the proportion of appropriate initial anti-biotherapy. Despite its use for the treatment of suspected or confirmed ESBL-E infections being controversial, PIP-TAZ was prescribed mainly as an empirical treatment. Ceftazidime–avibactam was the preferred non-carbapenem definitive treatment. Antibiotic stewardship should target new BL/BLI combinations.

## Figures and Tables

**Table 1:** Characteristics of critically ill patients with ESBL-E BSI.

Variable	Total (n = 185)
Demographics	
Age (years), median (IQR)	60 (19)
Male, n (%)	126 (68.0)
Underlying diseases, n (%)	
McCabe > 1	53 (28.6)
Diabetes mellitus	64 (34.6)
Heart failure	25 (13.5)
COPD	34 (18.4)
Chronic renal insufficiency	21 (11.4)
Immunodeficiency, n (%)	45 (24.3)
Immunosuppressive therapy	22 (11.9)
Solid cancer	22 (11.9)
Hematological malignancy	18 (9.7)
Transplantation	9 (4.9)
Admission, n (%)	
Medical	162 (87.6)
Unscheduled surgical	23 (12.4)
ESBL-E risk factors, n (%)	
Antibiotic treatment in the last 3 months	153 (82.7)
Colonization with ESBL-E	147 (79.5)
Hospital acquired infection	166 (89.7)
Hospital stay before BSI onset, days, median (IQR)	21 (24)
ICU stay before BSI onset, days, median (IQR)	15 (22)
Source of BSI, n (%)	
Pneumonia	103 (55.7)
Urinary tract	26 (14.1)
Catheter related	24 (13.0)
Intra-abdominal	24 (13.0)
Unknown	6 (3.2)
Disease severity at BSI onset, n (%)	
SOFA score, median (IQR)	6 (5)
Pitt score, median (IQR)	4 (4)
Shock	97 (52.4)
Mechanical ventilation	134 (72.4)
Clinical outcome, n (%)	
ICU stay, days, median (IQR)	35 (47)
In-ICU mortality	71 (38.4)

COPD: chronic obstructive pulmonary disease.

**Table 2:** Results of Enterobacteriaceae identifications and in vitro antimicrobial susceptibility testing *.

Antibiotics	Total n = 185	*Klebsiella* sp. n = 126	*Escherichia coli* n = 29	*Enterobacter* sp. n = 22	Others n = 8
Amoxicillin–clavulanate	19/185 (10)	9/126 (7)	10/29 (34)	0/22 (0)	1/8 (13)
Piperacillin–tazobactam	54/185 (29)	24/126 (19)	24/29 (83)	4/22 (18)	2/8 (25)
CMI < 8	25/185 (14)	7/126 (6)	17/29 (59)	0/22 (0)	1/8 (13)
Temocillin	47/83 (57)	26/53 (49)	12/19 (63)	9/10 (90)	0/1 (0)
Cefoxitin	83/159 (52)	69/115 (60)	14/17 (82)	0/20 (0)	0/7 (0)
Cefepime	59/185 (32)	43/126 (34)	4/29 (14)	9/22 (41)	3/8 (38)
Ertapenem	153/185 (83)	99/126 (76)	29/29 (100)	22/22 (100)	3/8 (38)
Ceftazidime–avibactam	114/120 (95)	88/93 (95)	7/8 (88)	14/14 (100)	5/5 (100)
Ceftolozan–tazobactam	64/109 (59)	47/83 (57)	6/6 (100)	7/15 (47)	4/5 (80)
Levofloxacin	54/185 (29)	32/126 (25)	11/29 (38)	8/22 (36)	3/8 (38)
Amikacin	159/185 (86)	111/126 (88)	28/29 (97)	18/22 (82)	2/8 (25)
Colistin	81/95 (85)	61/74 (82)	3/3 (100)	10/11(91)	7/7 (100)
Trimethoprim–sulfamethoxazole	40/185 (22)	23/126 (18)	13/29 (45)	1/22 (5)	3/8 (38)
Tigecyclin	81/95 (85)	61/74 (82)	3/3 (100)	10/11 (91)	7/7 (100)

* Results are expressed as the proportion of sensitive strains among the strains tested for the different antibiotics. Number of sensitive strains/number of tested strains (%).

**Table 3:** Univariate and multivariate analysis of the factors associated with an empirical prescription of a carbapenem in adequation with the guidelines.

Variables	Factors Associated with an Adequate Empirical Prescription of a Carbapenem			
	Univariate		Multivariate	
	OR (CI 95%)	*p*	OR (CI 95%)	*p*
Sex (Male)	0.744 [0.256–2.162]	0.587		
Age	0.975 [0.938–1.012]	0.186	0.979 [0.918–1.043]	0.506
Diabetes	1.647 [0.539–5.030]	0.381		
Chronic cardiac insufficiency	1.776 [0.368–8.575]	0.475		
Chronic respiratory insufficiency	0.328 [0.109–0.990]	0.048	0.551 [0.115–2.631]	0.455
Chronic renal insufficiency	2.250 [0.267–18.929]	0.456		
Cancer	0.584 [0.139–2.450]	0.463		
Hemopathy	2.250 [0.267–18.929]	0.456		
Immunodepression	1.989 [0.528–7.496]	0.310		
Antibiotic allergy	0.935 [0.098–8.879]	0.953		
Antibiotics within 3 months	1.083 [0.316–3.711]	0.899		
ESBL colonization	54.312 [10.192–289.417]	<0.0001	107.921 [9.303–1251.910]	0.0002
Length of stay in hospital	1.009 [0.987–1.031]	0.422		
SOFA at BSI onset	54.312 [10.192–289.417]	0.008	1.061 [0.787–1.431]	0.696
Septic shock	3.885 [1.334–11.313]	0.013	11.029 [0.936–129.888]	0.056
VM at BSI onset	0.874 [0.300–2.548]	0.805		
Respiratory source of BSI	3.152 [1.087–9.134]	0.035	3.456 [0.768–15.558]	0.106

OR: odds ratio; CI: confidence interval; VM: mechanical ventilation.

## Data Availability

Data are available from the corresponding author (contact: ameybeck@ch-tourcoing.fr).

## References

[1] Rodríguez-Baño J., Navarro M.D., Romero L., Muniain M.A., de Cueto M., Ríos M.J., Hernandez J.R., Pascual A. (2006). Bacteremia due to extended-spectrum beta-lactamase-producing *Escherichia coli* in the CTX-M era: A new clinical challenge. Clin. Infect. Dis..

[2] Tumbarello M., Sanguinetti M., Montuori E., Trecarichi E.M., Posteraro B., Fiori B., Citton R., D’Inzeo T., Fadda G., Cauda R. (2007). Predictors of mortality in patients with bloodstream infections caused by extended-spectrum beta-lactamase-producing Enterobacteriaceae: Importance of inadequate initial antimicrobial treatment. Antimicrob. Agents Chemother..

[3] Jean S.S., Harnod D., Hsueh P.R. (2022). Global threat of carbapenem-resistant Gram-negative bacteria. Front. Cell. Infect. Microbiol..

[4] Tamma P.D., Rodriguez J. (2017). The use of noncarbapenem β-lactams for the treatment of extended-spectrum β-lactamase infections. Clin. Infect. Dis..

[5] Harris P.N.A., Tambyah P.A., Lye D.C., Mo Y., Lee T.H., Yilmaz M., Alenazi T.H., Arabi Y., Falcone M., Bassetti M. (2018). MERINO Trial Investigators and the Australasian Society for Infectious Disease Clinical ResearchNetwork (ASID-CRN). Effect of piperacillin-tazobactam vs meropenem on 30-Day mortality for patients with *E. coli* or *Klebsiella pneumoniae* bloodstream infection and ceftriaxone resistance: A randomized clinical trial. JAMA.

[6] Antibiothérapie Des Infections à entérobactéries Et à *Pseudomonas aeruginosa* Chez L’adulte: Place Des Carbapénèmes Et de Leurs Alternatives. Mai 2019. Mise à Jour Mars 2023. https://has-sante.fr/jcms/c_2968915/fr/antibiotherapie-des-infections-a-enterobacteries-et-a-pseudomonas-aeruginosa-chez-l-adulte-place-des-carbapenemes-et-de-leurs-alternatives.

[7] European Committee on Antimicrobial Susceptibility Testing (EUCAST 2012). Breakpoint Tables for Interpretation of MICs and Zone Diameters. Version 2.0. http://www.eucast.org/fileadmin/src/media/PDFs/EUCAST-files/Breakpoint-tables/Breakpoint-table-v-2.0-120222.pdf.

[8] McCabe W.R., Jackson C.G. (1962). Gram-negative bacteremia: Etiology and ecology. Arch. Intern. Med..

[9] Le Gall J.R., Lemeshow S., Saulnier F. (1993). A new simplified acute physiology score (SAPS II) based on a European/North American multicenter study. JAMA.

[10] Vincent J.L., Moreno R., Takala J., Willatts S., De Mendonça A., Bruining H., Reinhart C.K., Suter P.M., Thijs L.G. (1996). The SOFA (Sepsis-related Organ Failure Assessment) score to describe organ dysfonction/failure. Intensive Care Med..

[11] Bone R.C., Fischer C.J., Clemmer T.P., Slotman G.J., Metz C.A., Balk R.A. (1989). The methylprednisolone severe sepsis study group. Sepsis syndrome: A valid clinical entity. Crit. Care Med..

[12] Weiss E., Zahar J.R., Lesprit P., Ruppe E., Leone M., Chastre J., Lucet J.C., Paugam-Burtz C., Brun-Buisson C., Timsit J.F. (2015). Elaboration of a consensual definition of de-escalation allowing a ranking of ß-lactams. Clin. Microbiol. Infect..

[13] Kern W.V., Rieg S. (2020). Burden of bacterial bloodstream infection-a brief update on epidemiology and significance of multidrug-resistant pathogens. Clin. Microbiol. Infect..

[14] McDanel J., Schweizer M., Crabb V., Nelson R., Samore M., Khader K., Blevins A.E., Diekema D., Chiang H.Y., Nair R. (2017). Incidence of Extended-Spectrum β-Lactamase (ESBL)-Producing *Escherichia coli* and *Klebsiella* Infections in the United States: A Systematic Literature Review. Infect. Control Hosp. Epidemiol..

[15] Lee C.H., Chu F.Y., Hsieh C.C., Hong M.Y., Chi C.H., Ko W.C., Lee C.C. (2017). A simple scoring algorithm predicting extended-spectrum β-lactamase producers in adults with community-onset monomicrobial Enterobacteriaceae bacteremia: Matters of frequent emergency department users. Medicine.

[16] Rottier W.C., Bamberg Y.R., Dorigo-Zetsma J.W., van der Linden P.D., Ammerlaan H.S., Bonten M.J. (2015). Predictive value of prior colonization and antibiotic use for third-generation cephalosporin-resistant enterobacteriaceae bacteremia in patients with sepsis. Clin. Infect. Dis..

[17] Madrid-Morales J., Sharma A., Reveles K., Velez-Mejia C., Hopkins T., Yang L., Walter E., Cadenaa J. (2021). Validation of Available Extended-Spectrum-Beta-Lactamase Clinical Scoring Models in Predicting Drug Resistance in Patients with Enteric Gram-Negative Bacteremia Treated at South Texas Veterans Health Care System. Antimicrob. Agents Chemother..

[18] Elligsen M., Pinto R., Leis J.A., Walker S.A.N., MacFaden D.R., Daneman N. (2021). Using prior culture results to improve initial empiric antibiotic prescribing: An evaluation of a simple clinical heuristic. Clin. Infect. Dis..

[19] Elligsen M., Pinto R., Leis J.A., Walker S.A.N., Daneman N., MacFadden D.R. (2021). Improving decision making in empiric antibiotic selection (IDEAS) for Gram-negative bacteremia: A prospective clinical implementation study. Clin. Infect. Dis..

[20] Tamma P.D., Aitken S.L., Bonomo R.A., Mathers A.J., van Duin D., Clancy C.J. (2022). Infectious Diseases Society of America 2022 Guidance on the Treatment of Extended-Spectrum β-lactamase Producing Enterobacterales (ESBL-E), Carbapenem-Resistant Enterobacterales (CRE), and Pseudomonas aeruginosa with Difficult-to-Treat Resistance (DTR-P. aeruginosa). Clin. Infect. Dis..

[21] Paul M., Carrara E., Retamar P., Tängden T., Bitterman R., Bonomo R.A., de Waele J., Daikos G.L., Akova M., Harbarth S. (2022). European Society of Clinical Microbiology and Infectious Diseases (ESCMID) guidelines for the treatment of infections caused by multidrug-resistant Gram-negative bacilli (endorsed by European society of intensive care medicine). Clin. Microbiol. Infect..

[22] Kadri S.S., Adjemian J., Lai Y.L., Spaulding A.B., Ricotta E., Prevots D.R., Palmore T.N., Rhee C., Klompas M., Dekker J.P. (2018). Difficult-to-treat resistance in Gram-negative bacteremia at 173 US Hospitals: Retrospective cohort analysis of prevalence, predictors, and outcome of resistance to all first-line agents. Clin. Infect. Dis..

[23] Teitelbaum D., Elligsen M., Katz K., Lam P.W., Lo J., MacFadden D., Vermeiren C., Daneman N. (2022). Introducing the escalation antibiogram: A simple tool to inform changes in empiric antimicrobials in the non responding patient. Clin. Infect. Dis..

[24] Guarascio A.J., Brickett L.M., Porter T.J., Lee N.D., Gorse E.E., Covey J.R. (2019). Developmment of a statewide antibiogram to assess regional trends in antibiotic-resistant ESKAPE organisms. J. Pharm. Pract..

[25] Huson M.A.M., Stolp S.M., van der Poll T., Grobusch P.M. (2014). Community-acquired bacterial bloodstream infections in HIV-infected patients: A systematic review. Clin. Infect. Dis..

[26] Lang R., Gill J.M., Viczko J., Naugler C., Church D. (2022). Risk factors and outcomes of bloodstream infections among people with human immunodeficiency virus: A longitudinal cohort study from 2000 to 2017. Open Forum Infect. Dis..

[27] Dellinger P., Rhodes A., Evans L., Alhazzani W., Beale R., Jaeschke R., Machado F.R., Masur H., Osborn T., Parker M.M. (2023). Surviving sepsis campaign. Crit. Care Med..

[28] Lien F., Lin H.S., Wu Y.T., Chiueh T.S. (2022). Bacteremia detection from complete blood count and differential leukocyte count with machine learning: Complementary and competitive with C-reactive protein and procalcitonin tests. BMC Infect. Dis..

[29] Li D., Li J., Zhao C., Liao X., Xie L., Shang W. (2023). Diagnostic value of procalcitonin, hypersensitive C-reactive protein and neutrophil-to-lymphocyte ratio for bloodstream infections in pediatric tumor patients. Clin. Chem. Lab. Med..

[30] Vincent J.L., Sakr Y., Singer M., Martin-Loeches I., Machado F.R., Marshall J.C., Finfer S., Pelosi P., Brazzi L., Aditianingsih D. (2020). Prevalence and outcomes of infection among patients in intensive care units in 2017. JAMA.

[31] Houard M., Rouze A., Ledoux G., Six S., Jaillette E., Poissy J., Preau S., Wallet F., Labreuche J., Nseir S. (2018). Relationship between digestive tract colonization and subsequent ventilator-associated pneumonia related to ESBL-producing Enterobacteriaceae. PLoS ONE.

[32] Barbier F., Bailly S., Schwebel C., Papazian L., Azoulay E., Kallel H., Siami S., Argaud L., Marcotte G., Misset B. (2018). Infection-related ventilator-associated complications in ICU patients colonised with extended-spectrum beta-lactamase-producing Enterobacteriaceae. Intensive Care Med..

[33] Das S., Li J., Riccobene T., Carrothers T.J., Newell P., Melnick D., Critchley I.A., Stone G.G., Nichols W.W. (2019). Dose Selection and Validation for Ceftazidime-Avibactam in Adults with Complicated Intra-Abdominal Infections, Complicated Urinary Tract Infections, and Nosocomial Pneumonia. Antimicrob. Agents Chemother..

[34] Isler B., Ezure Y., Garcia-Fogeda Romero J.L., Harris P., Stewart A.G., Paterson D.L. (2021). Is ceftazidime/avibactam an option for serious infections due to extended-spectrum-β-lactamase- and AmpC-producing enterobacterales?: A systematic review and meta-analysis. Antimicrob. Agents Chemother..

[35] Dequin P.F., Aubron C., Faure H., Garot D., Guillot M., Hamzaoui O., Lemiale V., Maizel J., Mootien J.Y., Osman D. (2023). The place of new antibiotics for Gram-negative bacterial infections in intensive care: Report of a consensus conference. Ann. Intensive Care.

[36] Zhang H., Xu J., Xiao Q., Wang Y., Wang J., Zhu M., Cai Y. (2023). Carbapenem-sparing beta-lactam/beta-lactamase inhibitors versus carbapenems for bloodstream infections caused by extended-spectrum beta-lactamase-producing Enterobacteriaceae: A systematic review and meta-analysis. Int. J. Infect. Dis..

[37] Umemura T., Kato H., Hagihara M., Hirai J., Yamagishi Y., Mikamo H. (2022). Efficacy of combination therapies for the treatment of multi-drug resistant Gram-negative bacterial infections based on meta-analyses. Antibiotics.

[38] Benetazzo L., Delannoy P.Y., Houard M., Wallet F., Lambiotte F., Vachée A., Batt C., Van Grunderbeeck N., Nseir S., Robineau O. (2020). Combination therapy with aminoglycoside in bacteremias due to ESBL-producing Enterobacteriaceae in ICU. Antibiotics.

[39] Kumar A., Zarychanski R., Light B., Parrillo J., Maki D., Simon D., Laporta D., Lapinsky S., Ellis P., Mirzanejad Y. (2010). Early combination antibiotic therapy yields improved survival compared with monotherapy in septic shock: A propensity-matched analysis. Crit. Care Med..

[40] Laterre P.F., Wittebole X., Van de Velde S., Muller A.E., Mouton J.W., Carryn S., Tulkens P.M., Dugernier T. (2015). Temocillin (6g daily) in critically ill patients: Continuous infusion versus three times daily administration. J. Antimicrob. Chemother..

[41] Marin-Candon A., Rosso-Fernandez C.M., Bustos de Godoy N., Lopez-Cerero L., Gutierrez-Gutierrez B., Lopez-Cortes L.E., Barrera Pulido L., BorregueroBorreguero I., Leon M.J., Merino V. (2021). Temocillin versus meropenem for the targeted treatment of bacteraemia due to third-generation cephalosporin-resistant Enterobacterales (ASTARTE): Protocol for a randomised, pragmatic trial. BMJ Open.

[42] Luyt C.E. Piperacillin-Tazobactam and Temocillin as Carbapenem-Alternatives for the Treatment of Severe Infections Due to Extended-Spectrum Beta-Lactamase Producing Gram-Negative Enterobacteriaceae in the Intensive Care Unit (PITAGORE). https://classic.clinicaltrials.gov/ct2/show/NCT05565222.

[43] Lo C.L., Lee C.C., Li C.W., Li M.C., Hsueh P.R., Lee N.Y., Ko W.C. (2017). Fluoroquinolone therapy for bloodstream infections caused by extended-spectrum beta-lactamase-producing *Escherichia coli* and *Klebsiella pneumoniae*. Microbiol. Immunol. Infect..

[44] Meije Y., Pigrau C., Fernández-Hidalgo N., Clemente M., Ortega L., Sanz X., Loureiro-Amigo J., Sierra M., Ayestarán A., Morales-Cartagena A. (2019). Non-intravenous carbapenem-sparing antibiotics for definitive treatment of bacteraemia due to Enterobacteriaceae producing extended-spectrum β-lactamase (ESBL) or AmpC β-lactamase: A propensity score study. Int. J. Antimicrob. Agents.

[45] Mission Spares. Surveillance de la Consommation des Antibiotiques et des Résistances Bactériennes en Établissement de Santé. Mission SPARES, Résultats 2021. Saint-Maurice: Santé Publique France, 2023. https://www.santepubliquefrance.fr/maladies-et-traumatismes/infections-associees-aux-soins-et-resistance-aux-antibiotiques/resistance-aux-antibiotiques/documents/enquetes-etudes/surveillance-de-la-consommation-des-antibiotiques-et-des-resistances-bacteriennes-en-etablissement-de-sante.mission-spares-resultats-2021.

[46] Bush K., Bradford P.A. (2020). Epidemiology of β-lactamase-producing pathogens. Clin. Microbiol. Rev..

[47] Zhu N.J., Rawson T.M., Mookerjee S., Price J.R., Davies F., Otter J., Aylin P., Hope R., Gilchrist M., Shersing Y. (2022). Changing patterns of bloodstream infections in the community and acute care across 2 coronavirus disease 2019 epidemic waves: A retrospective analysis using data linkage. Clin. Infect. Dis..

[48] Murphy C.N., Fowler R., Balada-Llasat J.M., Carroll A., Hanna Stone H., Akerele O., Buchan B., Windham S., Hopp A., Ronen S. (2020). Multicenter Evaluation of the BioFire FilmArray Pneumonia/Pneumonia Plus Panel for Detection and Quantification of Agents of Lower Respiratory Tract Infection. J. Clin. Microbiol..

[49] Strich J.R., Ricotta E., Warner S., Lai Y.L., Demirkale C.Y., Hohmann S.F., Rhee C., Klompas M., Palmore T., Powers III J.H. (2021). Pharmacoepidemiology of Ceftazidime-Avibactam Use: A Retrospective Cohort Analysis of 210 US Hospitals. Clin. Infect. Dis..

